# HIF-1α Protects Granulosa Cells From Hypoxia-Induced Apoptosis During Follicular Development by Inducing Autophagy

**DOI:** 10.3389/fcell.2021.631016

**Published:** 2021-01-22

**Authors:** Zonghao Tang, Renfeng Xu, Zhenghong Zhang, Congjian Shi, Yan Zhang, Hongqin Yang, Qingqiang Lin, Yiping Liu, Fengping Lin, Baorong Geng, Zhengchao Wang

**Affiliations:** ^1^Provincial Key Laboratory for Developmental Biology and Neurosciences, Provincial University Key Laboratory of Sport and Health Science, Key Laboratory of Optoelectronic Science and Technology for Medicine of Ministry of Education, College of Life Sciences, Fujian Normal University, Fuzhou, China; ^2^Key Laboratory of Medical Electrophysiology of Ministry of Education and Sichuan Province, Drug Discovery Research Center, Southwest Medical University, Luzhou, China

**Keywords:** hypoxia, autophagy, apoptosis, granulosa cell, follicular development

## Abstract

Owing to the avascular structure of the ovarian follicle, proliferation of granulosa cells (GCs) and development of follicles occur under hypoxia, which is obviously different from the cell survival requirements of most mammalian cells. We hypothesized that autophagy may exert an inhibitory effect on GC apoptosis. To decipher the underlying mechanism, we constructed a rat follicular development model using pregnant mare serum gonadotropin and a cell culture experiment in hypoxic conditions (3% O_2_). The present results showed that the autophagy level was obviously increased and was accompanied by the concomitant elevation of hypoxia inducible factor (HIF)-1α and BNIP3 (Bcl-2/adenovirus E1B 19kDa-interacting protein 3) in GCs during follicular development. The levels of Bax (Bcl2-associated X) and Bcl-2 (B-cell lymphoma-2) were increased, while the activation of caspase-3 exhibited no obvious changes during follicular development. However, inhibition of HIF-1α attenuated the increase in Bcl-2 and promoted the increase in Bax and cleaved caspase-3. Furthermore, we observed the downregulation of BNIP3 and the decrease in autophagy after treatment with a specific HIF-1α activity inhibitor (echinomycin), indicating that HIF-1α/BNIP3 was involved in autophagy regulation in GCs *in vivo*. In an *in vitro* study, we also found that hypoxia did not obviously promote GC apoptosis, while it significantly enhanced the activation of HIF-1α/BNIP3 and the induction of autophagy. Expectedly, this effect could be reversed by 3-methyladenine (3-MA) treatment. Taken together, these findings demonstrated that hypoxia drives the activation of HIF-1α/BNIP3 signaling, which induces an increase in autophagy, protecting GC from apoptosis during follicular development.

## Introduction

Oxygen is one of the major factors influencing the survival of mammalian cells, and the maintenance of oxygen tension within a physiological range is critical to ensure proper cell metabolism required for cellular functions (Zhang et al., 2011a, b, 2019a; Tang et al., 2017a, 2019). Therefore, in order to maintain normal supply of oxygen and nutrients, the developing tissues are ubiquitously accompanied by invasion of blood vessels. However, distinctively, capillary invasion is restricted to the outside of the follicle basement membrane, generating an avascular and hypoxic niche within follicles (Suzuki et al., 1998; Bianco et al., 2005; Zhang et al., 2019b). Expectedly, the proliferation of GCs and the expansion of follicles further exacerbate the imbalance of oxygen supply and consumption in ovarian follicles during follicular development (Fischer et al., 1992; Wang et al., 2012, 2015, 2017; Zhang et al., 2012; Wu et al., 2015, 2016). It has been demonstrated that the oxygen tension of follicular fluid in a small follicle is higher than that of large follicles and exhibits a lower trend during follicular maturation (Fischer et al., 1992; Zhang et al., 2012). However, the reason why GCs still maintain its proliferative phenotype under hypoxia conditions remains poorly understood.

Autophagy is an evolutionarily conserved catabolic mechanism, which envelopes the unnecessary components of proteins or organelles with a double layer membrane and transports the cargo into lysosomes for degradation (Mizushima and Levine, 2020). The findings of recent decades demonstrated that autophagy can serve as a self-protective mechanism under starvation, hypoxia, and many other stressful conditions (Galluzzi et al., 2014). Indeed, evidence has shown that autophagy is increased during mouse follicular development and plays a positive role in GC survival (Zhou et al., 2017). Consistently, previous investigations have also shown the increase in autophagy during rat follicle development, while suggesting that autophagy may exacerbate apoptosis during follicular development, as it exhibits a similar trend with the activation of caspase-3 (Choi et al., 2011, 2013). However, because the deficiency of reversing verification, it is possible that the activation of caspase-3 results from follicular atresia (Choi et al., 2013). Therefore, it is necessary to clarify the contribution of autophagy to follicular development in rats and the underlying mechanism.

HIF-1α is an important transcriptional factor that regulates the survival of mammalian cells under hypoxia conditions (Kumar and Choi, 2015). Activation of HIF-1α can orchestrate the expression of a large battery of downstream genes that reprogram cell metabolism. For example, upregulation of HIF-1α may convert the metabolism of mitochondria from oxidative phosphorylation into glycolysis to minimize the demand of oxygen supply (Cheng et al., 2014). Furthermore, HIF-1α can also modulate the induction of autophagy targeting redundant components for lysosomal degradation to avoid the skew of metabolic balance (Azad et al., 2008). Previously, our lab (Tang et al., 2017b) and others (Zhou et al., 2017) have illustrated the role of HIF-1α in autophagy regulation in mouse GCs. However, its role in rat GCs remains largely unknown. Particularly, there is controversy regarding the role of autophagy in GC apoptosis in mice and rats.

Thus, the present study utilized a follicular development rat model induced using pregnant mare serum gonadotropin (PMSG), and then determined the role of hypoxia in GC apoptosis. We hypothesized that hypoxia is an important factor involved in the increase of autophagy by inducing HIF-1α signaling, which prevents GC apoptosis. The present investigation aims to clarify the role of hypoxia in autophagy regulation and the underlying mechanism during follicular development in rats. To the best of our knowledge, this study elucidates the role of hypoxia in autophagy and apoptosis during rat follicular development for the first time, which may bridge the deficiency of our understanding on hypoxia-driven apoptosis in rat GCs.

## Materials and Methods

### Animals

In the present study, 3-week old female Sprague–Dawley rats were used to induce ovarian follicular development using PMSG (15 IU, i.p.) and the animals were maintained under 14-h light/10-h dark conditions for 2 days before treatment. To inhibit the activation of HIF-1α, echinomycin (Ech, 100 μg/kg) was used together with PMSG treatment. The animals were maintained under 14-h light/10-h dark conditions and executed via cervical dislocation at designated time points, including 0, 6, and 12 h after PMSG treatment. Animal experimentation protocols were reviewed and approved by the Ethics Committee on Animal Experimentation of Fujian Normal University. After removal of the connective tissues, the ovaries were washed with PBS (phosphate belanced solution) and immediately fixed in 4% paraformaldehyde (PFA) for histological processing or used for granulosa cell collection.

### Granulosa Cell Collection and Culture

For *in vitro* culture of GCs, ovarian samples from immature rats (3-week old) were harvested 48 h after PMSG (15 IU, i.p.) treatment (Chen et al., 2013). These ovarian samples were then placed in serum-free DMEM/F12 (Dulbecco’s modified eagle media/nutrient mixture F-12) medium. To release and collect granulosa cells (GCs), follicles were punctured by using a 25-gage needle. After that, GCs were suspended in DMEM/F12 medium (Hyclone) supplemented with 10% fetal bovine serum (Gibco), 100 mg/mL streptomycin sulfate (Hyclone), and 100 mg/mL penicillin G (Hyclone). For the hypoxia treatment, cells were maintained in a hypoxic incubator (3% O_2_, 5% CO_2_, 92% N_2_, 37°C). For the autophagy inhibition, GCs were incubated with or without 3% O_2_ and were treated with 3-MA (10 μM) for 12 h.

### Flow Cytometry

To evaluate the apoptosis of GCs under hypoxia, cells were digested with 0.25% trypsin (Hyclone) and washed with PBS. Then, GCs were stained with Annexin V- fluorescein isothiocyanate (FITC)/propidium iodide (PI) (Becton Dickinson) according to the instructions from the manufacturer. The apoptosis rates of GCs were analyzed using a BD FACScan system (Becton Dickinson, NJ, United States). Briefly, the Annexin V stained cells were counted and set as apoptotic cells, the apoptotic rate was calculated by three repeat experiments at least.

### ROS Level Detection

ROS (Reactive Oxygen Species) levels in rat GCs were measured by using a commercial kit (Beyotime) according to the protocol provided by the manufacturer. Briefly, ovarian samples were homogenized and centrifuged to collect the supernatant. Then, supernatants were seeded into 96 wells and incubated at 37°C for 20 min. After that, samples were measured at an excitation wavelength of 488 nm and emission wavelength of 525 nm.

### SOD Activity Examination

The superoxide dismutase (SOD) activity of ovarian samples was measured using a commercial kit (Nanjing jiancheng Bioengineering). To detect the SOD activity, ovarian samples were homogenized. Saline was used to generate a 10% tissue homogenate, and saline was set as the blank. Then, the tissue homogenate was centrifuged at 1,500 × *g* for 8 min to collect the supernatant of the ovarian sample. Similarly, the samples were then pipetted into 96 wells and incubated at 37°C for 30 min. Finally, the absorbances of the sample supernatants were measured at 450 nm using a microplate reader.

### Western Blotting

To analyze the expression of the proteins of interest, GCs were collected and lysed using ice-cold RIPA lysis buffer (Beyotime, P0013B) containing PMSF. After that, cell lysates were centrifugated (13,000 × *g*, 4°C, 30 min), and the supernatant of the lysates were collected. The protein concentration was measured by using a commercial kit (Beyotime, P0010). The whole lysates (40 μg/lane) were separated using 8 or 10% SDS–PAGE (dodecyl sulfate, sodium salt (SDS)-Polyacrylamide gel electrophoresis), and then transferred to a PVDF (polyvinylidene fluoride) membrane (Millipore, Bedford, MA, United States). To block non-specific binding, the membrane was blocked with 5% skim milk in TBST, and then incubated with the primary antibodies (Supplementary Table 1) overnight at 4°C. After washing with TBST for three times, the membrane was incubated with HRP (horseradish peroxidase)-conjugated secondary antibody at room temperature for 1 h. The immunoreactive bands were visualized using ECL (enhanced chemiluminescence) (Amersham Pharmacia Biotech) and an image analyzer (Bio-Rad). The expression values of bands were quantified by using ImageJ.

### Immunohistochemistry

After fixation with 4% PFA, ovarian samples were dehydrated with gradient ethylalcohol and then embedded with paraffin. The embedded samples were cut (5-μm slices), followed by dewaxing and rehydration. For antigen retrieval, the sections were placed in a steamer for 20 min in 10 mM citric buffer. After that, the sections were incubated with 3% H_2_O_2_ for 10 min to inhibit endogenous peroxide activity. Non-specific binding was blocked with 5% bovine serum albumin (BSA, Sigma), followed by treatment with anti-LC-3I/II (microtubule-associated protein 1 light chain-3I/II) polyclonal antibody (Abcam, ab48394, 1:300). After washing, sections were incubated with an HRP-labeled secondary antibody (ZSGB Bio) for 1h followed by PBS washing. After that, antibody complexes were visualized with diaminobenzidine tetrahydrochloride chromogen. Then, the sections were counterstained with hematoxylin, dehydrated, and mounted.

### Transmission Electron Microscopy

Granulosa cells were collected from the ovaries of immature rats (3-week old) after PMSG treatment for different time points. For transmission electron microscopy (TEM) observation, samples were prepared and fixed with 2.5% (vol/vol) glutaraldehyde (Solarbio, P1126) in PBS (4°C, pH 7.4, 0.1 M) for 24 h. After that, samples were then postfixed with 1% OsO_4_ (Ted Pella) for 1.5 h. Graded alcohol series were used for gradient dehydration, then samples were embedded in Araldite (SPI, 90529-77-4). Embedded tissues were sectioned to approximately 60 nm, and then mounted on formvar-coated grids (Ted Pella, 01700-F). The ultrathin sections were contrasted with 3% aqueous uranyl acetate and lead citrate staining. The sections were then examined and photographed under a transmission electron microscope (JEM-2100, Japan).

### Statistical Analysis

All experimental values are presented as mean±standard error (SE). The significant differences in the mean values within or between treatment groups were evaluated using one-way analysis of variance, followed by Tukey’s multiple range test. Statistical analysis was conducted using SPSS version 20. Statistically significant differences were inferred at a *P* < 0.05.

## Results

### Autophagy Is Increased During Follicular Development

**FIGURE 1:**
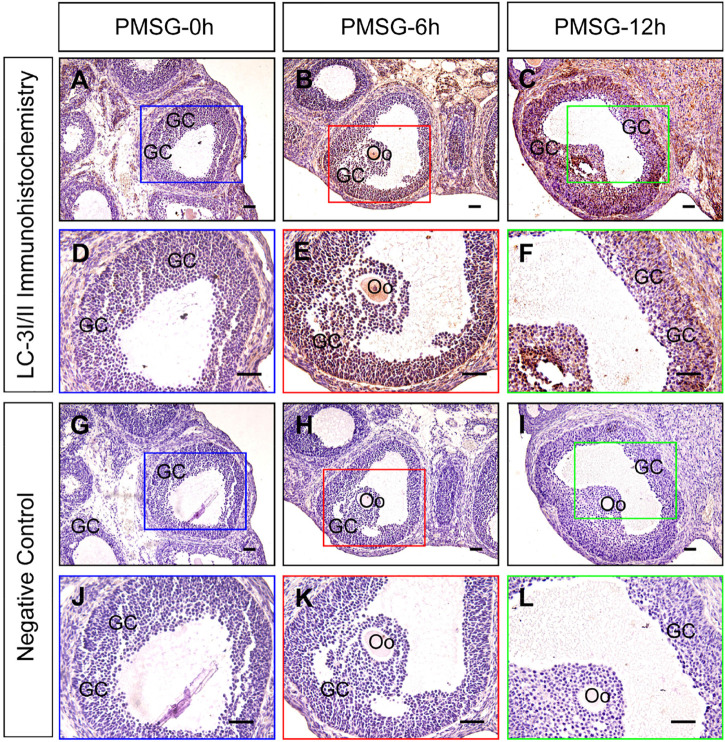
Immunohistochemical staining of LC-3I/II during follicular development. Three-week old female Sprague–Dawley rats were treated with PMSG to induce follicular development. LC-3I/II expression was examined using immunohistochemistry at 0, 6, and 12 h after PMSG treatment. In negative control, slides were incubated with serum instead of LC-3I/II antibody. GC, granulosa cell, Oo, oocyte, Scale bar = 100 μm.

**FIGURE 2:**
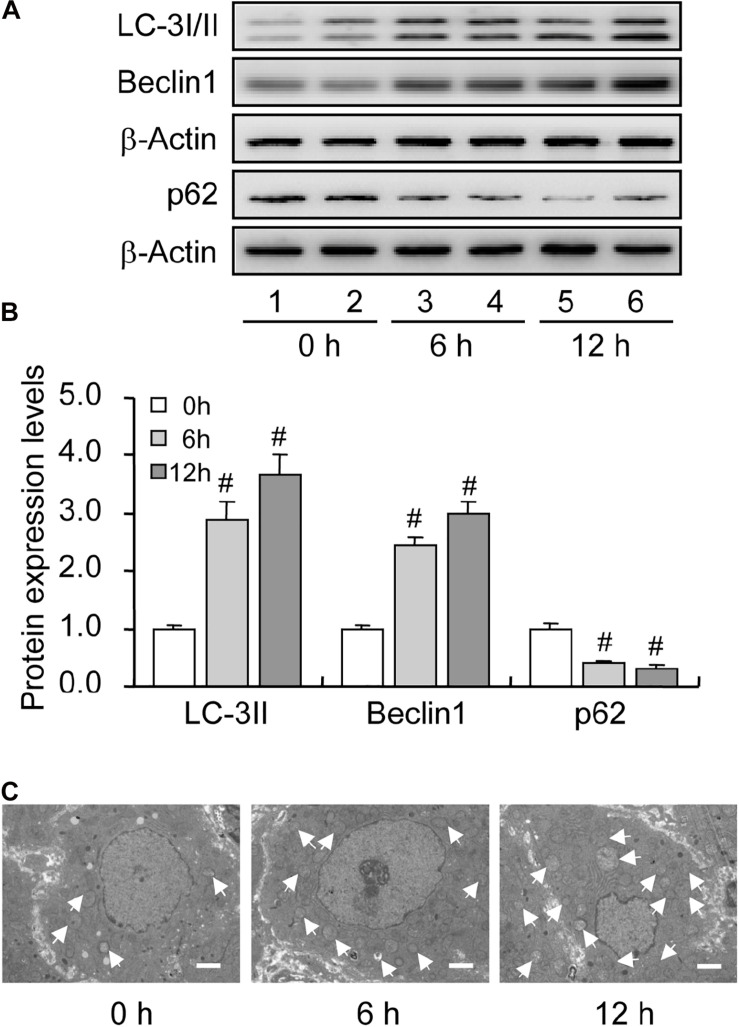
Expression changes in the level of autophagy-related proteins during follicular development. LC-3I/II, Beclin 1, and p62 expression was examined using western blotting at 0, 6, and 12 h after PMSG treatment. **(A)** Representative immunoblotting of LC-3I/II, Beclin 1, and p62. **(B)** Densitometric quantification of LC-3II, Beclin 1, and p62. **(C)** TEM observation of autophagosomes in the granulosa cells at 0, 6, and 12 h after PMSG treatment. White arrow indicates autophagosomes. Scale bar = 2 μm. Each value represents the mean ± SE. One-way analysis of variance (ANOVA) was used to analyze the data, followed by a Tukey’s multiple range test. *N* = 6. #, *P* < 0.05 vs. 0 h.

Previous investigations have demonstrated an increase in the autophagy level in rat GCs (Choi et al., 2010), and that the accumulation of autophagosomes is an important factor leading to GC apoptosis (Choi et al., 2011). To clarify the role of autophagy during this process, we treated immature rats with PMSG to induce follicular development. The results showed that autophagy marker protein LC-3I/II was mainly expressed in GCs and increased either 6 or 12 h after treatment (Figure 1). Furthermore, the expression of beclin 1 and LC-3II was also increased as evidenced by the result of Western blotting (Figures 2A,B). Conversely, the expression of p62, an essential receptor of autophagy, declined concomitantly, indicating the increase in autophagy flux during follicular development (Figures 2A,B), which was further confirmed by the result of TEM (Figure 2C). These results are consistent with that observed in mouse GCs (Zhou et al., 2017), further indicating the involvement of autophagy during rat follicular development.

### HIF-1α/BNIP3 Is Activated During Follicular Development

**FIGURE 3:**
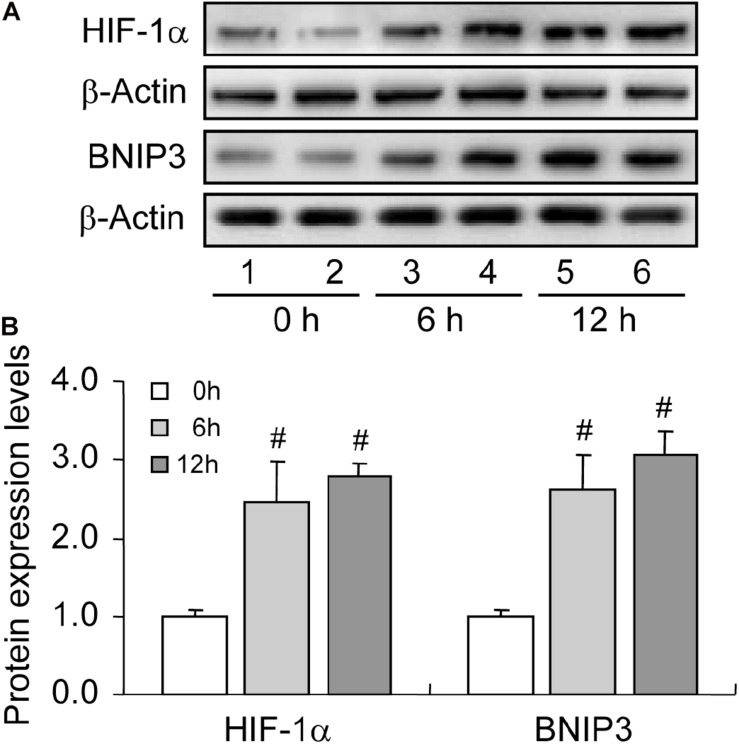
Expression changes of HIF-1α and BNIP3 during follicular development. HIF-1α and BNIP3 expression was examined using western blotting at 0, 6, and 12 h after PMSG treatment. **(A)** Representative immunoblotting of HIF-1α and BNIP3. **(B)** Densitometric quantification of HIF-1α and BNIP3. Each value represents the mean ± SE. One-way analysis of variance (ANOVA) was used to analyze the data, followed by a Tukey’s multiple range test. *N* = 6. #, *P* < 0.05, vs. 0 h.

HIF-1α is the primary regulator of mammalian cells under hypoxic conditions, exerting critical roles in cellular metabolism through the orchestration of downstream genes. Of note, HIF-1α can also protect mammalian cells from hypoxia-induced apoptosis by inducing autophagy (Zhou et al., 2017). Given the hypoxic environment within follicles, we further detected the expression of HIF-1α in rat GCs during follicular development. Expectedly, the expression levels of HIF-1α (Figures 3A,B) and its downstream protein BNIP3 (Figures 3A,B) increased during follicular development. These results implied that the activation of HIF-1α/BNIP3 may be upstream of autophagy regulation in GCs during follicular development.

### Increased Bcl-2 Protects GCs From Apoptosis During Follicular Development

**FIGURE 4:**
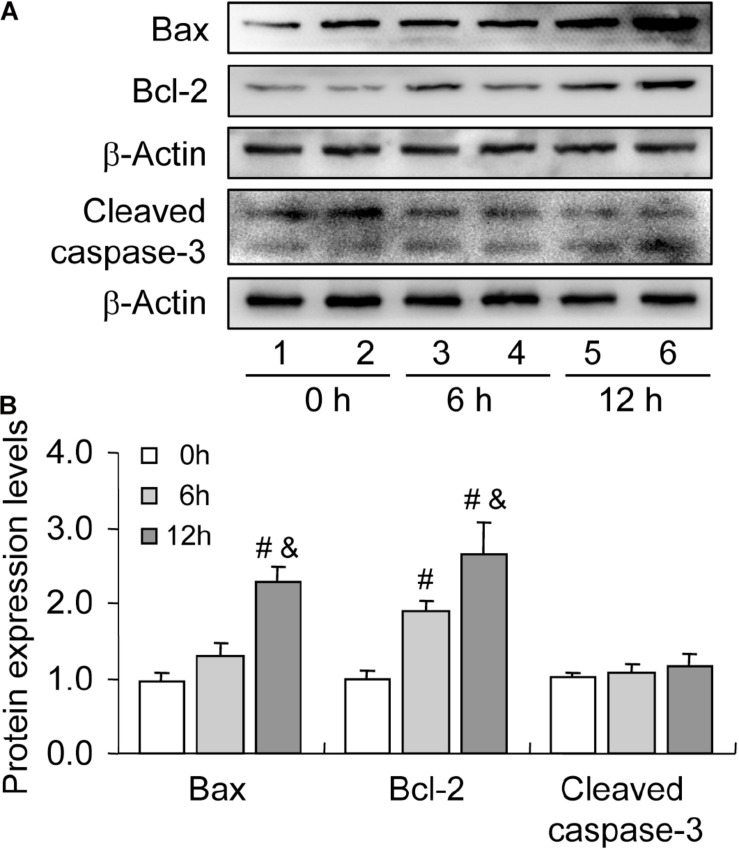
Expression changes of apoptosis-related proteins during follicular development. Bax, Bcl-2, and cleaved caspase-3 expression was examined using western blotting at 0, 6, and 12 h after PMSG treatment. **(A)** Representative immunoblotting of Bax, Bcl-2, and cleaved caspase-3. **(B)** Densitometric quantification of Bax, Bcl-2, and cleaved caspase-3. Each value represents the mean ± SE. One-way analysis of variance (ANOVA) was used to analyze the data, followed by a Tukey’s multiple range test. *N* = 6. #, *P* < 0.05, vs. 0 h. &, *P* < 0.05, vs. 12 h.

Bax is a member of the Bcl-2 family, which plays a positive role in programed cell death and in the maintenance of the Bax/Bcl-2 ratio, being important for cell survival (Brady and Gil-Gómez, 1998). Many pieces of evidence have demonstrated that severe hypoxia can obviously promote cell apoptosis by elevating the expression level of Bax (Tang et al., 2017a; Ge et al., 2018). To verify the involvement of Bax in cell apoptosis, we detected the expression of Bax during follicular development. The level of Bax was increased during follicular development (Figures 4A,B). However, we also observed the concomitant increase in Bcl-2 level during this process (Figures 4A,B). Therefore, we also detected the expression of cleaved caspase-3 and found no obvious changes during follicular development (Figures 4A,B). These findings suggested that hypoxia induces Bax expression, while elevated Bcl-2 level may exert a protective role in GC survival during follicular development.

### Inhibition of HIF-1α Suppresses BNIP3 Expression and Autophagy Level During Follicular Development

**FIGURE 5:**
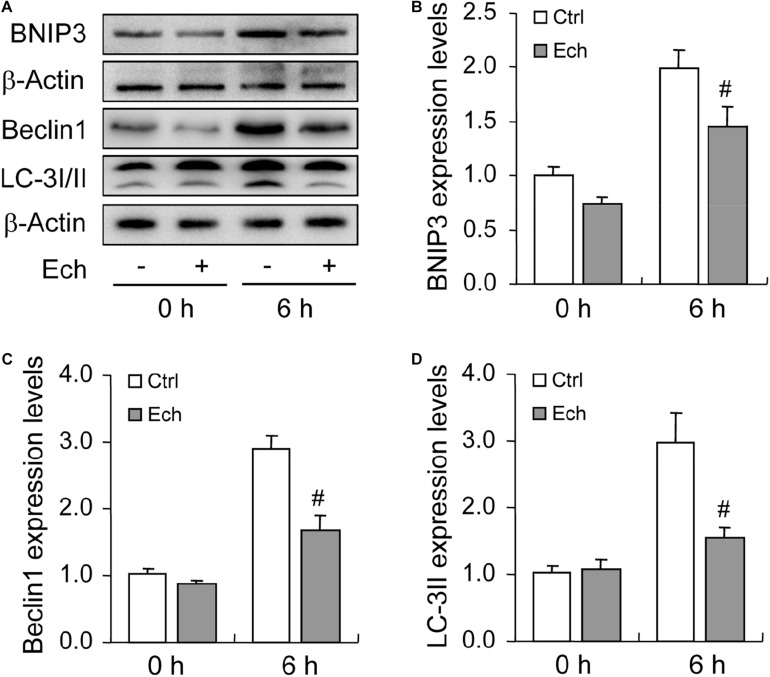
Effect of inhibited HIF-1α on autophagy-related protein expression during follicular development. HIF-1α was inhibited by HIF-1α specific inhibitor (Ech) for 6 h, and then the expression of BNIP3, Beclin 1, and LC-3I/II were examined using Western blotting. **(A)** Representative immunoblotting of BNIP3, Beclin 1, and LC-3I/II. **(B)** Densitometric quantification of BNIP3. **(C)** Densitometric quantification of Beclin 1. **(D)** Densitometric quantification of LC-3II. Each value represents the mean ± SE. One-way analysis of variance (ANOVA) was used to analyze the data, followed by a Tukey’s multiple range test. *N* = 6. #: *P* < 0.05, vs. Control.

To further verify the role of HIF-1α in BNIP3 expression and autophagy regulation, we inhibited HIF-1α activity by using a specific inhibitor Ech. Expectedly, inhibition of HIF-1α obviously declined the level of BNIP3 in GCs (Figures 5A,B), indicating that BNIP3 was regulated by HIF-1α in GCs during follicular development. Furthermore, we detected the compromise of the autophagy level, as evidenced by the downregulation of Beclin 1 (Figures 5A,C) and LC3-II (Figures 5A,D). These findings demonstrated that BNIP3 is downstream of HIF-1α, which may play an important role in autophagy regulation in GCs during follicular development.

### Inhibited HIF-1α Increases Oxidative Stress and Promotes Apoptosis During Follicular Development

**FIGURE 6:**
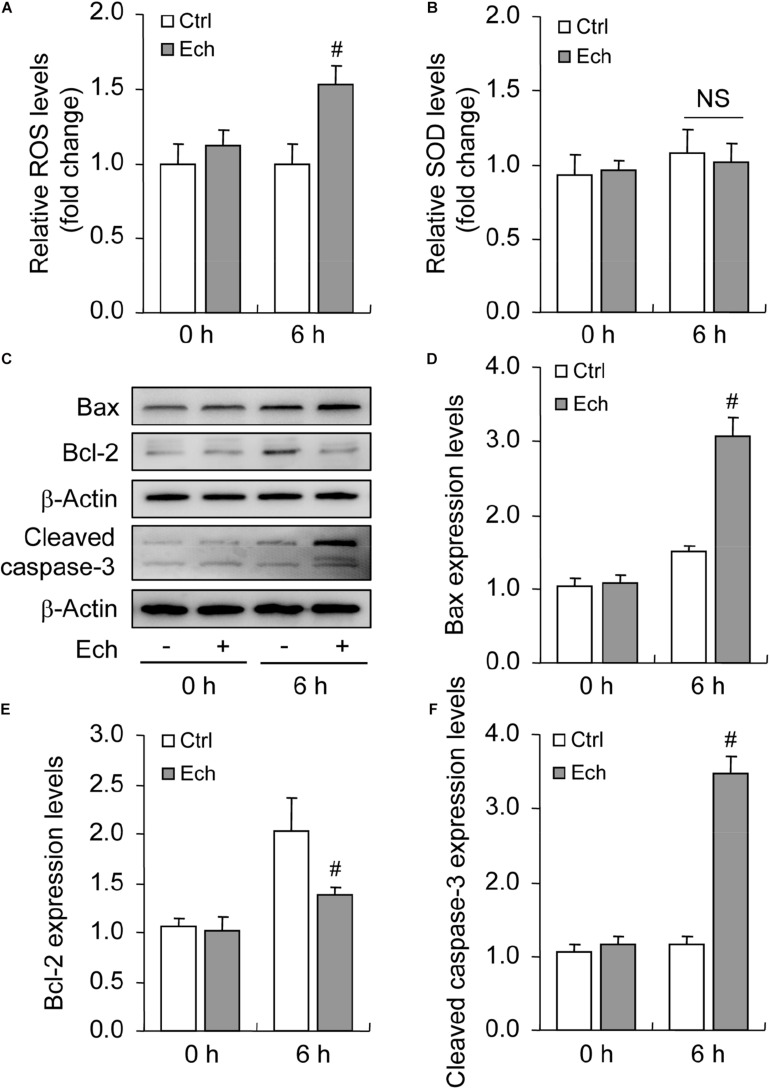
Effect of inhibited HIF-1α on ROS generation and GC apoptosis during follicular development. HIF-1α was inhibited by Ech for 6 h, and then ROS, SOD, Bax, Bcl-2, and cleaved caspase-3 were examined. **(A)** The level of ROS. **(B)** The level of SOD. **(C)** Representative immunoblotting of Bax, Bcl-2, and cleaved caspase-3. **(D)** Densitometric quantification of Bax. **(E)** Densitometric quantification of Bcl-2. **(F)** Densitometric quantification of cleaved caspase-3. Each value represents the mean ± SE. One-way analysis of variance (ANOVA) was used to analyze the data, followed by a Tukey’s multiple range test. *N* = 6. #, *P* < 0.05, vs. Control. Ech, echinomycin.

Oxidative stress is characterized by an imbalance of ROS generation and elimination, which are essential contributors for apoptosis under hypoxia (Fuhrmann and Brüne, 2017). To clarify the involvement of HIF-1α in oxidative stress, we detected the levels of both ROS (Figure 6A) and SOD (Figure 6B) after Ech treatment, and found that the inhibition of HIF-1α enhanced the generation of ROS (Figure 6A) in GCs during follicular development, while no obvious effect on the activity of SOD was detected (Figure 6B). Furthermore, we demonstrated that inhibition of HIF-1α decreased the level of Bcl-2 and fortified the expression of Bax compared with the control (Figures 6C–E). Also, Ech obviously elevated the expression of cleaved caspase-3 (Figures 6C,F). Morphological observation showed that Ech significantly stagnated the development of rat follicles as well (Supplementary Figures 1A,B). Taken together, these findings demonstrated that HIF-1α protects GCs from apoptosis by inhibiting oxidative stress and skewing Bax/Bcl-2 balance.

### Inhibited Autophagy Promotes Hypoxia-Induced Apoptosis During the Culture of GCs *in vitro*

**FIGURE 7:**
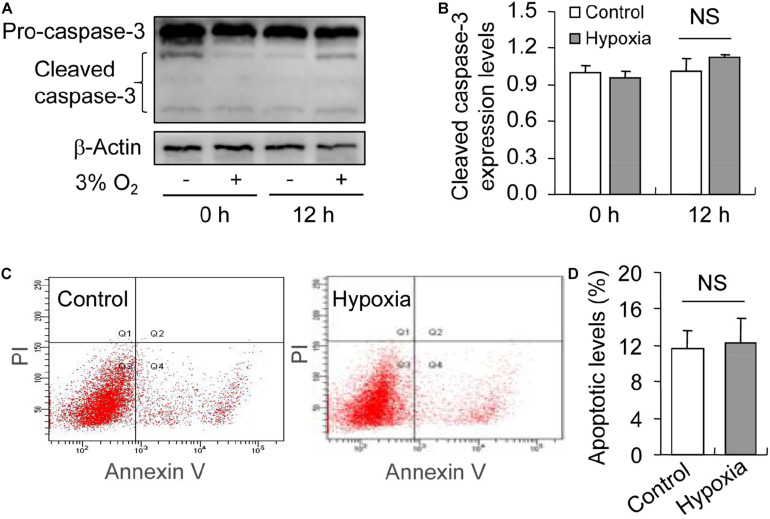
Effect of hypoxia on apoptosis of GCs cultured *in vitro*. GCs were cultured *in vitro* with and without hypoxia, and then apoptosis was examined. **(A)** Representative immunoblotting of caspase-3. **(B)** Densitometric quantification of cleaved caspase-3. **(C)** Representative images of flow cytometry using Annexin V-PI staining. **(D)** Percentages of GC apoptosis. Each value represents the mean ± SE. One-way analysis of variance (ANOVA) was used to analyze the data, followed by a Tukey’s multiple range test. *N* = 6. NS, no significance.

**FIGURE 8:**
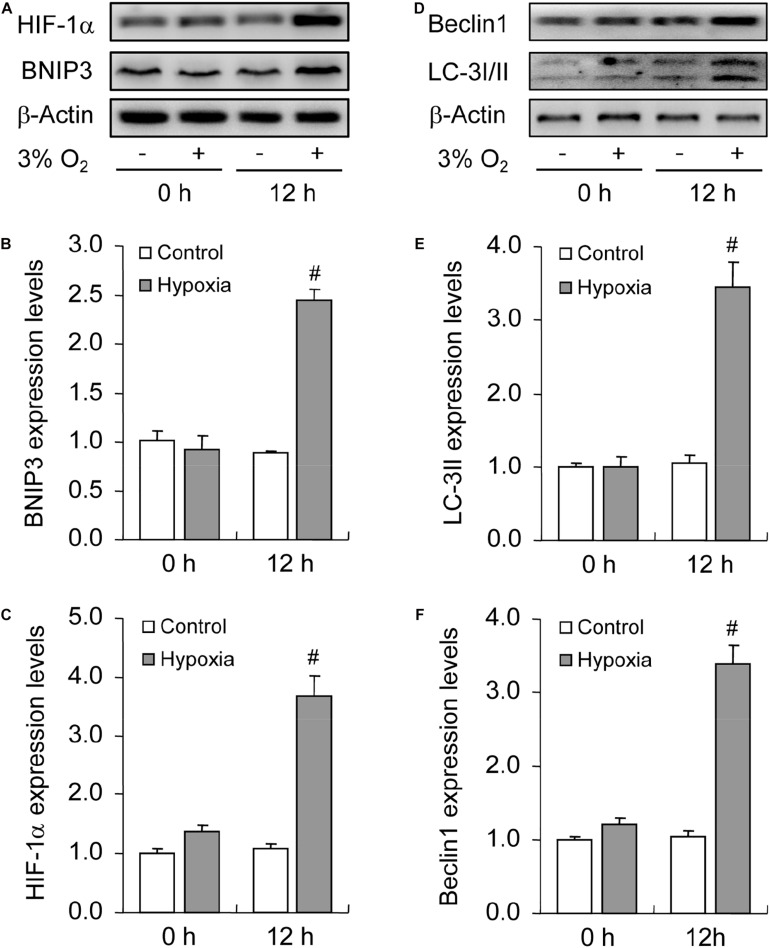
Effect of hypoxia on autophagy of GCs cultured *in vitro*. GCs were cultured *in vitro* with and without hypoxia, and then autophagy-related protein expression was examined. **(A)** Representative immunoblotting of HIF-1α and BNIP3. **(B)** Densitometric quantification of HIF-1α. **(C)** Densitometric quantification of BNIP3. **(D)** Representative immunoblotting of LC-3I/II and Beclin 1. **(E)** Densitometric quantification of LC-3II. **(F)** Densitometric quantification of Beclin 1. Each value represents the mean ± SE. One-way analysis of variance (ANOVA) was used to analyze the data, followed by a Tukey’s multiple range test. *N* = 6. #, *P* < 0.05, vs. Control.

**FIGURE 9:**
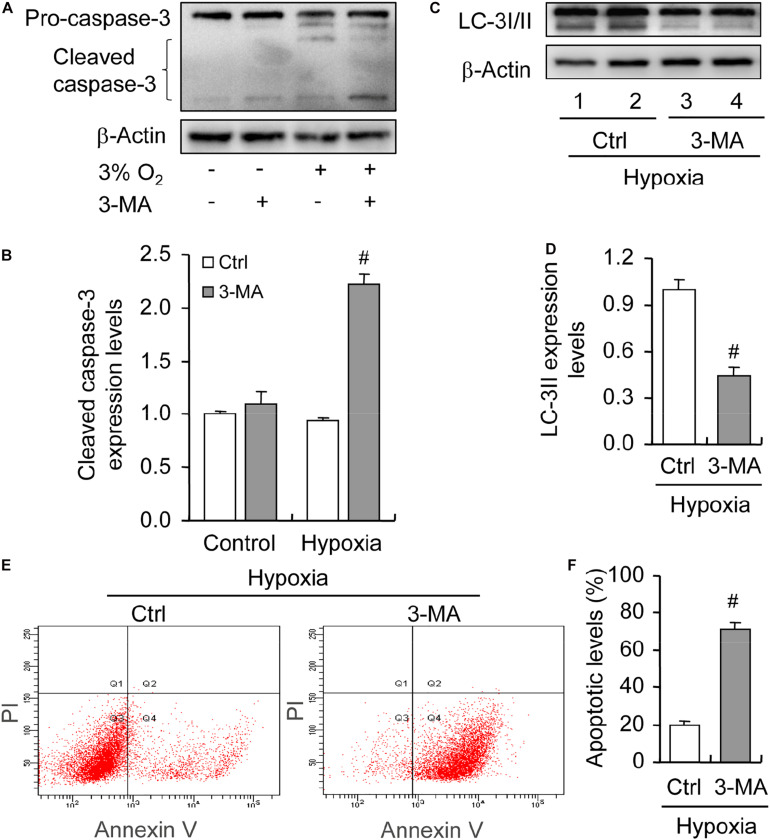
Effect of autophagy inhibition on apoptosis of GCs cultured *in vitro* with and without hypoxia. GCs were cultured *in vitro* with hypoxia, autophagy was inhibited by 3-MA, and then cleaved caspase-3 expression was examined. **(A)** Representative immunoblotting of caspase-3. **(B)** Densitometric quantification of cleaved caspase-3. **(C)** Representative immunoblotting of LC-3I/II. **(D)** Densitometric quantification of LC-3II. **(E)** Representative images of flow cytometry using Annexin V-PI staining. **(F)** Percentages of GC apoptosis. Each value represents the mean ± SE. One-way analysis of variance (ANOVA) was used to analyze the data, followed by a Tukey’s multiple range test. *N* = 6. #, *P* < 0.05, vs. Control.

To illustrate the role of hypoxia in apoptosis of rat GCs, we isolated GCs and cultured them under 3% O_2_. The results indicated that hypoxia treatment for 12 h did not fortify the activation of caspase-3 in GCs (Figures 7A,B). This conclusion was further verified by the flow cytometry results (Figures 7C,D). In addition, we observed the increase in the levels of HIF-1α and BNIP3 (Figures 8A–C) in rat GCs under hypoxia, as well as in LC-3II and Beclin 1 (Figures 8D–F), further indicating the contribution of hypoxia and HIF-1*α*/BNIP3 pathway to autophagy regulation in rat GCs. To further determine the effect of autophagy on the survival of rat GCs, we inhibited the induction of autophagy using 3-MA. The results indicated that autophagy inhibition (Figures 9C,D) facilitated the activation of caspase-3 in rat GCs (Figures 9A,B) and increased their apoptotic levels (Figures 9E,F). Therefore, these findings demonstrated that HIF-1*α*/BNIP3 is a critical pathway involved in the regulation of autophagy, which encumber hypoxia-induced caspase-3 activation by inducing autophagy in rat GCs.

## Discussion

Oxygen is one of the primary factors required for mammalian cell survival, which can obviously affect the metabolism and function of cells, especially when the oxygen tension is decreased. The deficiency of vascular invasion generates an avascular niche in rat follicles, which enables the development of GCs under hypoxia. Autophagy is a stress driven pro-survival mechanism in eukaryocytes, and previous studies from our lab (Tang et al., 2017b) and other labs (Zhou et al., 2017) have demonstrated the role of HIF-1α in autophagy of mouse GCs. Recent findings also indicated that HIF-1α plays an important role in the survival of porcine GCs under hypoxia (Li et al., 2020). However, hitherto, few studies have elucidated the underlying survival mechanism of rat GCs under hypoxia. Here, we demonstrated that autophagy is increased during rat follicular development, which is regulated in a HIF-1α/BNIP3-dependent manner and exerts a protective effect on GC survival. Consistently, these findings were further verified by *in vitro* experiments.

Because of the deficiency of vascular invasion, the ovarian follicle is a hypoxic core in mammalian ovaries. We inferred that there might exist an anti-apoptotic mechanism in rat GCs to counteract hypoxia-induced apoptosis during follicular development (Wang et al., 2015, 2017; Wu et al., 2015, 2016). Indeed, previous investigations have demonstrated that autophagy exhibits an increasing trend during follicular development in both mouse (Zhou et al., 2017) and rat (Choi et al., 2010). In recent years, some investigations demonstrated that autophagy can protect mouse GCs from apoptosis by attenuating mitochondrial impairment, and that autophagy inhibition decreases mitochondrial potential (Zhou et al., 2017). These findings indicated that autophagy is a self-protection mechanism of mitochondrial balance in mouse GCs (Zhou et al., 2017). Consistently, a recent study has also demonstrated the positive role of autophagy in mitochondrial quality control in porcine GCs (Li et al., 2020). However, although previous studies have elucidated the changes in autophagy level during rat follicular development and suggested that autophagy may exert a positive role on rat GC apoptosis, instead of inhibition (Choi et al., 2010), this is inconsistent with what was observed in mice. Nevertheless, more evidence is required to corroborate this conclusion, as they did not inhibit autophagy in rat GCs (Choi et al., 2010). In recent years, most studies have used mouse models, which has suspended this contradiction until now. In the present study, we showed that the autophagy level was concomitantly increased during rat follicular development and protected GCs from apoptosis by ameliorating the oxidative stress and skewing Bax/Bcl-2 balance in GCs. Expectedly, these findings are in parallel with those in mouse GCs, indicating that autophagy may function as a self-protective mechanism during mammalian GC development by maintaining mitochondrial homeostasis.

HIF-1α is a master regulator of cell metabolism under hypoxia. Previous investigations have shown that HIF-1α plays essential roles in GC survival and follicular development in both mouse (Zhou et al., 2017) and rat (Kim et al., 2009). Kim et al. (2009) demonstrated that inhibition of HIF-1α by Ech obviously impeded hCG-induced ovulation in mice. In the present study, we also showed that Ech clearly stagnated the development of rat follicles. Furthermore, inhibition of HIF-1α made a significant impact on GCs, namely decreasing the autophagy level and increasing the apoptotic level. These findings demonstrated that HIF-1α is involved in the induction of autophagy in rat GCs and exerts a protective effect on GC survival. To the best of our knowledge, this is the first study to elucidate the role of HIF-1α in autophagy during rat follicular development, which can provide us a better understanding of the role of HIF-1α in GC survival and follicular development.

It has been demonstrated that FSH (follicle-stimulating hormone), an essential sexual hormone facilitating GC proliferation and follicular development, is a critical regulator of HIF-1α activation in GCs, which prevents the loss of mitochondrial balance by inducing HIF-1α-mediated mitophagy in mouse (Zhou et al., 2017) and porcine GCs (Li et al., 2020). Consistently, we also observed the increase of oxidative stress after HIF-1α inhibition. However, it is now unclear whether hypoxia is an essential factor involved in HIF-1α activation and GC apoptosis during rat follicular development. Based on the best available parameter estimates, Redding et al. (2008) suggested that the mean dissolved oxygen levels in human follicular fluid range between 1.5 and 6.7%, which is similar with those in bovine follicles (Baddela et al., 2018). In the present study, we incubated rat GCs under hypoxic conditions (3.0%), which is consistent with the *in vivo* conditions and found that hypoxia did not obviously increase the apoptosis of rat GCs. These finding were in line with our previous hypothesis that there may exist a self-protective mechanism in GCs to exempt them from hypoxia-induced cell apoptosis.

Furthermore, we detected the obvious activation of HIF-1α/BNIP3 signaling and an increase in the autophagy level in rat GCs under hypoxia, and inhibition of autophagy led to the elevation of GC apoptosis (∼3.5-fold) as evidenced by the result of flow cytometry analysis. Therefore, we concluded that HIF-1α can protect GCs from hypoxia-induced apoptosis by inducing autophagy. Nevertheless, it is also noteworthy that recent evidence has indicated that the apoptosis of porcine GCs is significantly increased under 1.0% O_2_ (Li et al., 2020). We inferred that this discrepancy may result from two aspects: the extent of hypoxia and the difference in cell types. To some extent, 1.0% O_2_ is more severe than normal physiology in mammalian ovaries, such as human (Redding et al., 2008) and bovine ovaries (Baddela et al., 2018). Of note, the activation of HIF-1α was also observed in porcine GCs, which protected mitochondria from impairment by inducing mitophagy in a PINK1 (PTEN induced putative kinase 1)-dependent manner (Li et al., 2020). Taken together, these findings demonstrated that the expression of HIF-1α induced by hypoxia is an adaptive mechanism for GC survival in mammalian follicles, even if there may exist some mechanism divergence.

In summary, our present study demonstrated that autophagy is increased during rat follicular development, which is regulated in a HIF-1α/BNIP3-dependent manner. The increase of autophagy is a self-protective mechanism of rat GCs under hypoxic conditions to prevent the skew of oxidative stress.

## Data Availability Statement

The original contributions presented in the study are included in the article/Supplementary Material, further inquiries can be directed to the corresponding author/s.

## Ethics Statement

The animal study was reviewed and approved by the Ethics Committee on Animal Experimentation of Fujian Normal University.

## Author Contributions

ZT, RX, ZZ, BG, and ZW conceived the work and wrote the manuscript. ZT, RX, ZZ, CS, YZ, HY, QL, YL, and FL performed the experiments and contributed the reagents, materials, and analysis tools. ZT, RX, ZZ, CS, YZ, HY, QL, YL, FL, BG, and ZW analyzed the data. All authors reviewed and approved the manuscript.

## Conflict of Interest

The authors declare that the research was conducted in the absence of any commercial or financial relationships that could be construed as a potential conflict of interest.
