# Supplementary material for: HIF-1α Protects Granulosa Cells From Hypoxia-Induced Apoptosis During Follicular Development by Inducing Autophagy
Source: Front Cell Dev Biol. 2021 Jan 22;9:631016. doi: 10.3389/fcell.2021.631016 (PMC7862574; doi:10.3389/fcell.2021.631016)
Supplement: Supplementary file 1 [file Table_1.DOC]

HIF-1α Protects Granulosa Cells from Hypoxia-Induced Apoptosis during Follicular Development by Inducing Autophagy

Zonghao Tang, Renfeng Xu, Zhenghong Zhang, Congjian Shi, Yan Zhang, Hongqin Yang, Qingqiang Lin, Yiping Liu, Fengping Lin, Baorong Gen, and Zhengchao Wang

Supplementary Material

# Supplementary Figures and Tables

## Supplementary Figures


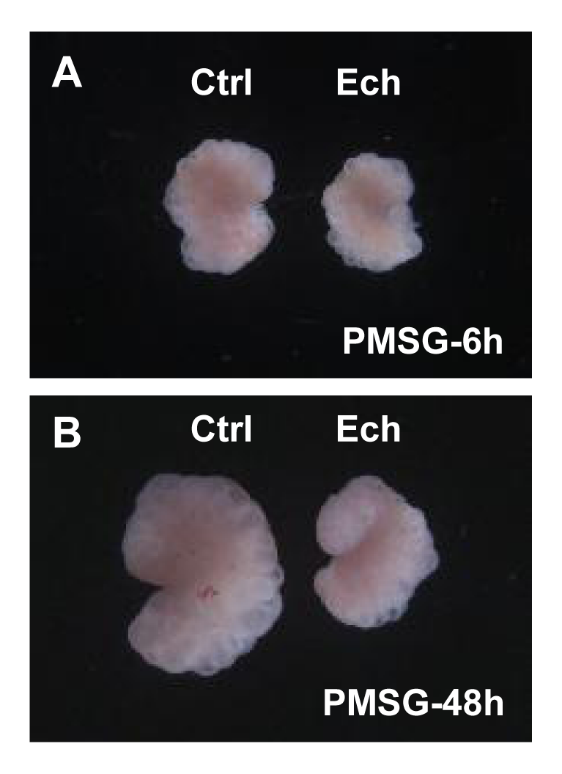


**Supplementary Figure 1.** **Effect of inhibited HIF-1α on follicular development.** Three-week old female Sprague–Dawley rats were treated with PMSG to induce follicular development, and HIF-1α was inhibited by Ech for 6 and 48 h. (A): Morphology of rat ovary at 6 h after Ech treatment. (B): Morphology of rat ovary at 48 h after Ech treatment. The magnification is 40×. Ech: echinomycin.

## Supplementary Tables

**Supplementary Table S**1 Antibody informations for western blotting

| Antibody Name | Company and City | Dilution Degree |
| --- | --- | --- |
| LC-3I/II | Abcam, Cambridge, MA, USA | 1:1000 |
| Beclin1 | Protein Tech Group, Wuhan, China | 1:2000 |
| β-actin | Protein Tech Group, Wuhan, China | 1:5000 |
| p62 | Abcam, Cambridge, MA, USA | 1:1000 |
| Caspase-3 | Protein Tech Group, Wuhan, China | 1:500 |
| cleaved caspase-3 | Cell Signaling Technology, Boston, MA, USA | 1:1000 |
| Bax | Protein Tech Group, Wuhan, China | 1:500 |
| Bcl-2 | Cell Signaling Technology, Boston, MA, USA | 1:1000 |
| HIF-1α | Santa Cruz Biotechnology, Dallas, TX, USA | 1:500 |
| BNIP3 | Abcam, Cambridge, MA, USA | 1:1000 |
| Goat anti-Mouse IgG | Beyotime Institute of Biotechnology, Haimen, China | 1:5000 |
| Goat anti-Rabbit IgG | Beyotime Institute of Biotechnology, Haimen, China | 1:5000 |
